# Clinical relevance of gemstone spectral CT in the diagnosis of carotid atherosclerosis

**DOI:** 10.3892/etm.2019.8077

**Published:** 2019-10-04

**Authors:** Jing-Jian Wang, Shu-Jie Fan, Long-Long Wang, Yan-Zhong Gao, Xiao-Juan Liu

Exp Ther Med 13: 2629-2636, 2017; DOI: 10.3892/etm.2017.4342

An interested reader drew to our attention the fact that a couple of figures that appeared in the above article had already appeared in the following paper (Fan Z-x, Wang L, Wang X-w, Li T-p, Yu S-j, Yu S and Su M: Gemstone spectral imaging combined with serum hypersensitive C-reactive protein, high mobility group box-1 protein preliminary study on carotid atherosclerosis plaque. Chin J Clin Neurosci 23: 266–371, 2015). Essentially, Figs. 3 and 4 in the Exp Ther Med paper had appeared as Figs. 1 and 2, respectively, in the Chin J Clin Neurosci article.

Following an investigation, the Journal was able to confirm that this duplication of the research data had occurred. On those grounds, the Editor of *Experimental and Theraputic Medicine* has decided that the above paper should be retracted. We were unable to make contact with the authors of the article published in *Experimental and Therapeutic Medicine*, despite every effort to do so. The Editor deeply regrets any inconvenience that this retraction has caused to the authors of the previously published paper, and to the readership of the Journal.

